# Validation of Factors Associated with Difficult Emergency Transport in Tokyo Before and After COVID-19 Pandemic: A Retrospective Observational Study

**DOI:** 10.14789/ejmj.JMJ25-0035-OA

**Published:** 2026-04-22

**Authors:** IPPEI OSUGI, DAISUKE USUDA, TOMOTARI INAMI, MASASHI KATO, ERI NAKAJIMA, RUNA SHIMIZU, TAKAYUKI KOMATSU, KEIKO MIZUNO, HIROKI TAKAMI, TOMOHISA NOMURA, MANABU SUGITA

**Affiliations:** 1 Department of Emergency and Critical Care Medicine, Juntendo University Nerima Hospital, Tokyo, Japan; 1 Department of Emergency and Critical Care Medicine, Juntendo University Nerima Hospital, Tokyo, Japan; 2 Department of Sports Medicine, Faculty of Medicine, Juntendo University, Tokyo, Japan; 2 Department of Sports Medicine, Faculty of Medicine, Juntendo University, Tokyo, Japan

**Keywords:** emergency medical services, difficult emergency transport, Tokyo, Tokyo Rule, Coronavirus disease 2019

## Abstract

**Objectives:**

To clarify and compare factors of difficult emergency transport before and after COVID-19 in Tokyo.

**Materials and Methods:**

We conducted a retrospective observation study. Data was collected by reviewing electronic medical records, and all patients transferred to our hospital as Tokyo Rule (TR) cases between April 1, 2010, and March 31, 2022, were enrolled. After enrollment, baseline, demographic, clinical characteristics of patients, and prehospital information about emergency medical services (EMS) activity time were collected. The primary outcome was defined as 30-day death. Firstly, we defined pre-COVID-19 group as being before January 15, 2020, and COVID-19 group afterwards. To predict factor for death, a univariate and multivariable logistic-regression analysis were applied to each parameter to identify predictors of death.

**Results:**

Total of 3,988 individuals were included. Of them, 2,005 were in pre-COVID-19 group, and 1,983 in COVID-19 group. In the univariate analysis, age, weekends and holidays, transportation time, respiratory rate, comorbidity of hypertension, and major diagnostic category were associated with death in pre-COVID-19 group. A similar situation was found for age, weekends and holidays, pulse oximetry, respiratory symptoms, and comorbidity of respiratory disease in COVID-19 group. In logistic regression analysis, independent predictors included weekends and holidays, comorbidity of hypertension, and major diagnostic category of gynecology in pre-COVID-19 group and weekends and holidays, respiratory symptoms, and comorbidity of respiratory disease in COVID-19 group.

**Conclusions:**

Factors of TR cases have changed since the COVID-19 pandemic.

## Introduction

### The emergency medical system setting in Japan

Japan uses a pre-hospital triage system built on a combination of urgency and the severity of patient condition^1)^. In Japan, emergency medical services (EMSs) that are considered part of fire departments can be summoned by calling the emergency number 119, and emergency medical technicians (EMTs) use a protocol to triage patients, based on recognizable acute symptoms^1)^. If a patient can be treated for their condition in the outpatient department, a designated primary emergency hospital will be selected; however, in the event that a patient’s condition necessitates hospitalization, a designated secondary emergency hospital is selected, and if admission to an ’intensive-care unit or an emergency operating room surgery is necessary, a designated tertiary emergency hospital will be selected^1)^. “Selection time” is defined as the length of time it takes for the EMTs to choose a designated emergency hospital, and to decide on how to transport the patient^1)^. If a hospital is unable to accept a patient, the EMS team will then need to choose a different destination hospital for the patient, leading to a longer selection time^2)^.

### Recent trends in emergency transport in Japan

In Japan, both the number of emergency transport cases and the number of difficult cases of emergency transport have been increasing from year to year^2)^. Based on this, the Tokyo Metropolitan Government enacted a guideline on August 1, 2009: these difficult emergency transport cases were deemed “Tokyo Rule (TR),” and specified hospitals were temporarily expected to accept ambulances^3)^. The definition of TR case is shown in Table 1. Our institution proactively accepts patients transferred by ambulance, including TR cases.

On the other hand, coronavirus disease 2019 (COVID-19) has had a marked effect on EMSs, when providing services both to patients with COVID-19 and without^1, 4, 5)^. The COVID-19 pandemic has had ramifications on various social systems, and EMS and transportation systems were among these^1, 6-8)^. This made it harder to maintain Japan’s conventional medical system^9)^. Compared to the pre-pandemic era, the post-COVID-19 era has seen longer times from arrival at the scene to patient contact, longer times spent at the scene, and longer total EMS activity times, especially when helping febrile patients^1, 10-12)^. However, the specifics of the situation, including details of change and etiology, remain unknown. The purpose of this study is to clarify and compare factors associated with difficult emergency transport in Tokyo before and after COVID- 19 pandemic, with the goal of improving the quality of emergency medicine in this area.

**Table 1:** The definition of TR case

1. Clinical condition of patients: Generally moderate or mild
2. Five or more medical institutions refused for ambulance transfer request of patient Twenty minutes have passed since the selection of medical institutions by emergency medical technician

If the case meets criteria "1 plus either of 2", the case is regarded as TR case. TR: Tokyo Rule

## Materials and Methods

### Study design

We conducted a retrospective observation study at Juntendo University Nerima Hospital (a 490-bed community hospital in Nerima City, Tokyo, Japan). Verbal and written informed consent were unnecessary. This study was approved by the ethics committee at Juntendo University Nerima Hospital (approval number: E22-0471), and the study was carried out according to the principles of the Declaration of Helsinki.

### Data collection

Data were collected by reviewing the electronic medical records and diagnosis procedure combination data maintained at our hospital, and all patients transferred to our hospital as TR cases between April 1, 2010, and March 31, 2022, were enrolled in the study. After enrollment, the following information was collected: age, gender, comorbidities (hypertension, diabetes, dyslipidemia, dialysis, psychiatric disease, cardiac disease, respiratory disease, stroke, and dementia), major diagnostic category (MDC) (neurology, orthopedic, or gynecology), background factors that could make transport difficult (post-drinking, trauma, living alone, receiving welfare, or foreign nationality), weekdays or weekends and holidays, transportation time (0:00-7:59, 8:00-15:59, or 16:00-23:59), EMS's activity time, and outcome (ward admission, intensive care unit (ICU) admission, or return home). In addition, the following information was also collected upon arrival at the emergency department: vital signs, state of consciousness (Japan Coma Scale), heart rate, systolic blood pressure, diastolic blood pressure, temperature, respiratory rate, and presence of respiratory symptoms. Here, we defined the pre-COVID-19 group as being before January 15, 2020, when the first COVID-19 patient was reported in Japan, and the COVID-19 group as being since January 15, 2020. We also defined the EMS's activity time as from detection of emergency calls to acceptance of patients at hospital. Furthermore, we defined patients who were transferred to other hospitals after their initial medical examination as being survivors. The inclusion criteria were all TR cases, and the exclusion criteria were cases lacking any parameters.

### Statistical analysis

The primary outcome was defined as 30-day death. Categorical variables are expressed as numbers and percentages, and continuous variables as means and standard deviations. The normal distribution of the continuous variables was assessed using the Kolmogorov-Smirnov test. We applied a univariate analysis to compare these parameters between the pre-COVID-19 and COVID-19 groups. For the comparison between the survival group and non- survival group, the potential confounding variables were entered into the univariate model, and factors that were significant in the univariate analysis were entered into multivariate analysis to adjust for confounding factors. In the univariate analysis, Student’s t-test or the Mann-Whitney U test was used for continuous variables, and the χ^2^ or Fisher exact test was used for categorical variables. A logistic regression analysis was applied to identify predictors of death in multivariate analysis.

The statistical analyses were performed using the STATA^®^ software package (version 10; STATA Corp LP). A statistical two-tailed significance level of 0.05 was used, and all hypothesis testing was also two-tailed.

## Results

### Baseline characteristics

**Figure 1:**
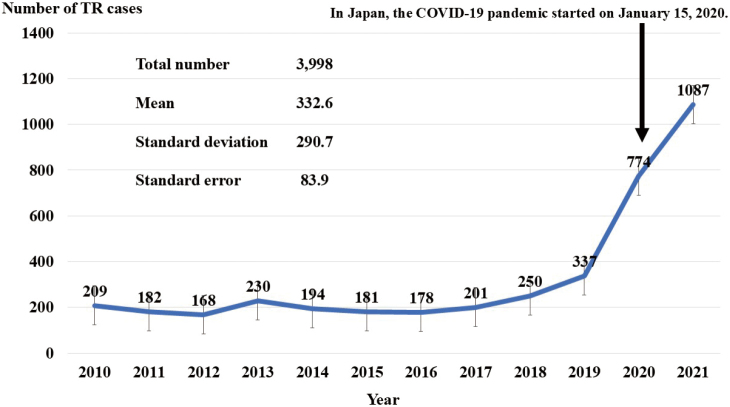
Annual number of TR cases transported to our hospital TR: Tokyo Rule

The annual number of TR cases at our hospital is shown in Figure 1. During the research period, a total of 4,109 patients were transferred to our hospital. Of these, 121 were excluded due to lack of any parameters, and 3,988 were included in the study. In Kolmogorov-Smirnov test, all continuous variables followed normal distribution. No statistical differences were found between the 121 excluded patients and 3,988 included patients. For the 3,988 patients included, 2,005 were in the pre-COVID-19 group, and 1,983 were in the COVID-19 group. Regarding outcomes, the number of deaths within 30 days was 10 (mortality rate of 0.5%) for the pre-COVID-19 group, and 22 (mortality rate of 1.1%) for the COVID-19 group. Baseline, demographic, and clinical characteristics and outcome data were obtained for all patients (Table 2). When comparing the pre-COVID-19 group with the COVID-19 group for each variable, the significant differences were confirmed for some variables. Of them, the following were significantly increased after COVID-19 pandemic: age, transportation time (8:00-15:59), heart rate, body temperature, respiratory symptoms, and comorbidities: hypertension, diabetes, dyslipidemia, cardiovascular disease, dementia, EMS's activity time, outcome (ward admission), and death within 30 days. In addition, the following were significantly decreased after COVID- 19 pandemic: transportation time (0:00-7:59), diastolic blood pressure, pulse oximetry, and comorbidities: psychiatric disease, MDCs (neurological or orthopedic), drinking-related aspects, welfare, and outcome (return home).

**Table 2:** Baseline, demographic, and clinical characteristics for patients in the pre-COVID-19 and COVID-19 groups

Parameter	pre-COVID-19 (n = 2005)	COVID-19 (n = 1983)	*p*-value
Males	1042 (52)	1015 (51)	0.62
Age (years)	62.3 ± 23.0	70.4 ± 22.6	< 0.01
Weekends and holidays	715 (36)	696 (35)	0.71
Transportation time: 0:00-7:59	619 (31)	495 (25)	< 0.01
8:00-15:59	488 (24)	623 (31)	< 0.01
16:00-23:59	898 (45)	865 (44)	0.46
Heart rate (beats/min)	90.5 ± 19	93.3 ± 19	< 0.01
Systolic blood pressure (mmHg)	132.9 ± 28.3	132.6 ± 26.8	0.68
Diastolic blood pressure (mmHg)	75.1 ± 16.9	73.4 ± 16.4	< 0.01
Body temperature (°C)	36.7 ± 0.9	37.2 ± 1.1	< 0.01
Respiratory rate (breaths/min)	19.7 ± 3.6	19.9 ± 3.5	0.09
Pulse oximetry (%)	95.7 ± 4.7	94.4 ± 6.4	< 0.01
Respiratory symptoms	218 (11)	433 (22)	< 0.01
Comorbidity: Hypertension	507 (25)	688 (35)	< 0.01
Diabetes	243 (12)	287 (14)	0.03
Dyslipidemia	80 (4)	141 (7)	< 0.01
Dialysis	47 (2)	49 (2)	0.79
Psychiatric disease	477 (24)	265 (13)	< 0.01
Cardiovascular disease	237 (12)	322 (16)	< 0.01
Respiratory disease	184 (9)	190 (10)	0.66
Dementia	212 (11)	333 (17)	< 0.01
MDC: Neurology	283 (14)	166 (8)	< 0.01
Orthopedic	405 (20)	345 (17)	0.02
Gynecology	20 (1)	12 (1)	0.17
Drinking related	211 (11)	94 (5)	< 0.01
Welfare	358 (18)	218 (11)	< 0.01
Trauma	292 (15)	179 (9)	0.66
Foreigner	19 (1)	21 (1)	0.72
EMS's activity time (min)	75.7 ± 25.9	80.4 ± 31.1	< 0.01
Outcome: Ward admission	887 (44)	1024 (52)	< 0.01
ICU admission	50 (2)	52 (3)	0.80
Return home	1050 (52)	881 (44)	< 0.01
COVID-19 positive	N/A	68 (3)	N/A
Death within 30-day	10 (0)	22 (1)	0.03

Data are presented as mean ± standard deviation (SD) or No. (%) as appropriate. COVID-19: Coronavirus disease 2019, MDC: Major diagnostic category, EMS: Emergency medical services, ICU: Intensive care unit

### Prediction of death in analysis

In the univariate analysis, age, weekends and holidays, transportation time (8:00-15:59 or 16:00-23:59), respiratory rate, comorbidity of hypertension, and MDC of gynecology were associated with death in the pre-COVID-19 group (Table 3). A similar situation was found for age, weekends and holidays, pulse oximetry, respiratory symptoms, and comorbidity of respiratory disease in the COVID-19 group (Table 4). Multivariate logistic regression analysis, weekends and holidays, comorbidity of hypertension, and MDC of gynecology remained independent predictors of death in the pre-COVID-19 group (Table 5). A similar situation was found for weekends and holidays, respiratory symptoms, and comorbidity of respiratory disease in the COVID-19 group (Table 6). Here, regarding the multivariate logistic regression analysis in the COVID-19 group, when age was dichotomized as ≥ 65 years versus < 65 years and entered into the multivariate logistic regression analysis, all patients aged ≥ 65 years belonged to the mortality group; therefore, age was excluded from the analysis.

**Table 3:** Prediction of death in the univariate analysis: pre-COVID-19 group

Parameter	Survivors (n = 1995)	Non-survivors (n = 10)	*p*-value
Males	1038 (52)	4 (40)	0.45
Age (years)	62.2 ± 23.0	77.7 ± 12.2	0.03
Weekends and holidays	706 (35)	9 (90)	< 0.01
Time: 0:00-7:59	617 (31)	2 (20)	0.46
8:00-15:59	481 (24)	7 (70)	< 0.01
16:00-23:59	897 (45)	1 (10)	0.03
Heart rate (beats/min)	90.5 ± 19.0	97 ± 21.4	0.28
Systolic blood pressure (mmHg)	132.9 ± 28.3	144 ± 31.1	0.22
Diastolic blood pressure (mmHg)	75.1 ± 16.9	72.2 ± 16.8	0.58
Body temperature (°C)	36.7 ± 0.9	36.9 ± 0.9	0.45
Respiratory rate (breaths/min)	19.7 ± 3.6	22.8 ± 7.9	< 0.01
Pulse oximetry (%)	95.7 ± 4.7	93 ± 9.3	0.07
Respiratory symptoms	215 (11)	3 (30)	0.05
Hypertension	500 (25)	7 (70)	< 0.01
Diabetes	241 (12)	2 (20)	0.44
Dyslipidemia	79 (4)	1 (10)	0.33
Dialysis	46 (2)	1 (10)	0.11
Psychiatric disease	476 (24)	1 (10)	0.31
Cardiovascular disease	235 (12)	2 (20)	0.42
Respiratory disease	183 (9)	1 (10)	0.93
Dementia	212 (11)	0 (0)	0.12
MDC: Neurology	283 (14)	0 (0)	0.20
Orthopedic	404 (20)	1 (10)	0.42
Gynecology	19 (1)	1 (10)	< 0.01
Drinking related	210 (11)	1 (10)	0.96
Welfare	357 (18)	1 (10)	0.52
Trauma	292 (15)	0 (0)	0.19
Foreigner	19 (1)	0 (0)	0.76
EMS's activity time (min)	75.7 ± 26.0	65.3 ± 19.3	0.10
30-day mortality	0.5%		

Data are presented as mean ± SD or No. (%) as appropriate. COVID-19: Coronavirus disease 2019, MDC: Major diagnostic category, EMS: Emergency medical services

**Table 4:** Prediction of death in the univariate analysis: COVID-19 group

Parameter	Survivors (n = 1961)	Non-survivors (n = 22)	*p*-value
Males	959 (49)	9 (41)	0.46
Age (years)	70.2 ± 22.6	84.8 ± 9.87	< 0.01
Weekends and holidays	693 (35)	3 (14)	0.03
Transportation time: 0:00-7:59	491 (25)	4 (18)	0.46
Transportation time: 8:00-15:59	616 (31)	7 (32)	0.97
Transportation time: 16:00-23:59	854 (44)	11 (50)	0.54
Heart rate (beats / min)	93.4 ± 19.0	92.5 ± 17.8	0.83
Systolic blood pressure (mmHg)	132.6 ± 26.8	129.9 ± 28.8	0.64
Diastolic blood pressure (mmHg)	73.4 ± 16.4	72.8 ± 18.4	0.88
Body temperature (°C)	37.2 ± 1.1	36.8 ± 0.8	0.08
Respiratory rate (breaths / min)	19.9 ± 3.5	20.2 ± 3.0	0.75
Pulse oximetry (%)	94.4 ± 6.4	90.1 ± 8.7	< 0.01
Respiratory symptoms	420 (21)	13 (59)	< 0.01
Comorbidity: Hypertension	680 (35)	8 (36)	0.87
Diabetes	284 (14)	3 (14)	0.91
Dyslipidemia	139 (7)	2 (9)	0.72
Dialysis	48 (2)	1 (5)	0.53
Psychiatric disease	163 (8)	2 (9)	0.54
Cardiovascular disease	316 (16)	6 (27)	0.16
Respiratory disease	182 (9)	8 (36)	< 0.01
Dementia	327 (17)	6 (27)	0.19
MDC: Neurology	164 (8)	2 (9)	0.90
Orthopedic	342 (17)	3 (14)	0.64
Gynecology	12 (1)	0 (0)	0.71
Drinking related	94 (5)	0 (0)	0.29
Welfare	218 (11)	0 (0)	0.10
Trauma	176 (9)	3 (14)	0.95
Foreigner	21 (1)	0 (0)	0.63
EMS's activity time (min)	77.2 ± 22.1	71.8 ± 9.9	0.14
30-day mortality	1.1%		

Data are presented as mean ± SD or No. (%) as appropriate. COVID-19: Coronavirus disease 2019, MDC: Major diagnostic category, EMS: Emergency medical services

**Table 5:** Prediction of death in the multivariate logistic regression analysis: pre-COVID-19 group

Parameter	OR	95% CI	*p*-value
Age (years)	1.97	0.28-13.74	0.49
Weekends and holidays	15.09	1.86-122.14	0.01
Time: 8:00-15:59	2.92	0.57-15.01	0.20
16:00-23:59	0.19	0.01-2.46	0.20
Respiratory rate (breaths/min)	1.74	0.4-7.56	0.46
Respiratory symptoms	2.41	0.49-11.95	0.28
Comorbidity: Hypertension	7.69	1.57-37.6	0.01
MDC: Gynecology	40.21	2.81-575.49	0.01
Age (years)	1.97	0.28-13.74	0.49

OR: Odds ratio, CI: Confidence interval, MDC: Major diagnostic category.

**Table 6:** Prediction of death in the multivariate logistic regression analysis: COVID-19 group

Parameter	OR	95% CI	*p*-value
Weekends and holidays	0.28	0.08-0.97	0.04
Pulse oximetry (%)	1.38	0.52-3.70	0.52
Respiratory symptoms	3.86	1.46-10.20	< 0.01
Comorbidity: Respiratory disease	3.75	1.49-9.44	< 0.01

OR: Odds ratio, CI: Confidence interval.

## Discussion

This is the first study to clarify and compare factors associated with difficult emergency transport before and after COVID-19 pandemic in Tokyo, Japan. Therefore, this article has value to report. The first COVID-19 case in Tokyo was confirmed on January 24, 2020, and the first wave in Japan began in February 2020. In this study, we pragmatically set the cutoff date as January 15, 2020. Because there is no substantial difference between these three dates, we consider this cutoff to be reasonable.

We report seven major findings. First, the factors of TR cases have changed since the COVID-19 pandemic. Second, the characteristics of the patients transferred to the hospital due to TR cases have also changed since the COVID-19 pandemic. More specifically, patients who were older, with more severe cases, and who had comorbidities have tended to be more likely to experience TR cases since the COVID-19 pandemic. In addition, patients with COVID-19-related symptoms have tended to be more likely to experience TR cases since the COVID-19 pandemic. Third, interestingly, however, neurology and orthopedic patients have not seen an increased tendency to experience TR cases since the COVID-19 pandemic. This finding is not compatible with previous reports and thus constitutes new knowledge^13)^. Fourth, the mortality rate of patients transferred to hospitals as TR cases has increased since the COVID-19 pandemic. Fifth, the cause of TR cases increased and various factors including of COVID-19 related vital signs as well as symptoms were led to TR cases after COVID-19 pandemic started. On the other hand, interestingly, the unrelated factors were also led to TR cases during COVID-19 pandemic. Sixth, EMS's activity time became longer since the COVID-19 pandemic. Seventh, cause of the death among patients transferred to the hospital due to TR cases have changed since the COVID-19 pandemic, especially COVID- 19 related symptoms were led to death after COVID-19 pandemic started.

We consider the reasons for these findings. Regarding the reason for the first finding, it was compatible with previous reports, since patients with symptoms related to COVID-19 have experienced longer selection times, leading to TR cases^1)^. In addition, in Japan, only limited medical institutions were expected to received and accepted COVID-19 patients or patients with symptoms related to COVID-19. Here, the basic demographic information related to TR cases about emergency transport in our target medical district is shown as Table 7^14-18)^. The number of people who were infected with COVID-19 was going up, and these patients tended to TR cases. Regarding the reason for the second and seventh finding, frailty, combined with certain comorbidities, played a significant role in predicting mortality among geriatric COVID-19 patients^19)^. We considered that these patients tended to call an ambulance, and then it led to TR cases combined with factors mentioned above. On the other hand, strict social distancing might be a contributing factor to the frequently undiscovered stroke cases among elderly living alone^5, 20)^. In fact, the negative correlation between lockdown and stroke admissions in Catalonia, Spain, was striking, with zero stroke admissions to a major stroke center during COVID-19 peak days^5, 21)^. For the same reason, regarding the reason for the third finding, we consider the orthopedic patients have not seen an increased tendency to experience TR cases since the COVID-19 pandemic. On the other hand, children and other younger patients consistently saw no correlation with difficult-to-transfer cases, even during the COVID-19 pandemic^22)^. On the other hand, since the COVID-19 pandemic, neurological and orthopedic patients have seen no tendency toward increased TR cases, and there was no correlation between gynecology patients and difficult-to-transfer cases. Regarding the fourth finding, we considered this reflected the increase of patients of COVID-19 or with symptoms related to COVID-19. These patients tended to TR cases. Regarding the fifth finding, we considered this was reflected in the reason for the shortage of the number of available ambulances, which are not only due to responding to the overwhelming EMS calls, but also to getting ready for the next call^5)^. Regarding the reason for sixth findings, the suspected reason was that the increased number of enquiries regarding emergency destinations for febrile patients during the COVID-19 pandemic period may have contributed to the extended selection time, implying that more time was required for patients to receive appropriate EMS at the hospital^1)^. Therefore, our results may suggest a burden on the EMS system imposed on both patients and EMTs due to the COVID-19 pandemic^1)^. In addition, these results will contribute to improving the quality of emergency medicine in this area.

Several limitations of this study merit consideration. First, we conducted only a single-center study. Therefore, these results only reflects the characteristics of the emergency medical care system in that specific area. Consequently, it is difficult to generalize the findings of this study to other regions in Tokyo or to Japan as a whole. Second, we failed to collect data on hospitalization periods for hospitalized patients, nor on patients transferred to a different hospital after their initial medical examination. Consequently, we were unable to perform multivariable analysis to determine the factors that lead to TR cases, and the results should be interpreted with caution. Third, we inquired with the Fire and Disaster Management Agency Ministry of Internal Affairs and Communications regarding the “time required for emergency medical services to select a medical institution,” but the information was not available. Therefore, we were unable to quantitatively analyze the “selection time” variable that directly defines TR cases. Fourth, we should have evaluated the magnitude of the change for each parameter, to clarify the etiology of TR cases changes. Fifth, we failed to collect data for cause of trauma or diseases. Therefore, we were unable to explain the causal factor of TR cases clearly. Sixth, a multivariable model was constructed despite the very small number of death events, particularly in the pre-COVID-19 group (n = 10); therefore, this approach is considered statistically weak. Seventh, in the multivariable logistic regression analysis of the pre-COVID-19 group, the 95% confidence interval were extremely wide. This is likely due to instability resulting from the small sample size.

**Table 7:** Basic information related to TR about emergency transport in our target medical district

Parameter (Unit)	Number	Date (As of)	Reference number
Number of populations	13,794,933	July 2023	18
Area wide (km^2^)	2,200	April 2024	19
Hospital's number	629	October 2022	20
TR hospital's number	89	April 2023	21
EMS's received calling (calls)	No data	N/A	N/A
EMS dispatched number (calls)	708,695 (TR 51,670, 7.3%)	October 2022	22

TR: Tokyo Rule, EMS: Emergency medical services.

## Conclusion

The factors of TR cases have changed since the COVID-19 pandemic. Patients who were older, with respiratory symptoms or hypoxia, and who had a comorbidity of respiratory disease have tended to be more likely to experience difficult emergency transport cases since the COVID-19 pandemic. These results will contribute to improving the quality of emergency medical systems and healthcare cooperation for the threat of future pandemics.

## Abbreviations

TR; Tokyo Rule, COVID-19; Coronavirus disease 2019, EMS; Emergency medical services, MDC; Major diagnostic category, ICU; Intensive care unit, SD; Standard deviation

## Author contributions

Concept and design: IO, TK, TM, MS. Acquisition, analysis, or interpretation of data: IO, DU, TI, MK, EN, RS, MS. Drafting of the manuscript: IO, DU, KM, HT. Critical review of the manuscript for important intellectual content: DU, TK, TN, MS. Supervision: TM, MS. All authors have reviewed the final version to be published and agreed to be accountable for all aspects of the work.

## Conflicts of interest statement

The authors declare that there are no conflicts of interest.
